# Supplementary material for: Protocol Optimization for Direct Reprogramming of Primary Human Fibroblast into Induced Striatal Neurons
Source: Int J Mol Sci. 2023 Apr 5;24(7):6799. doi: 10.3390/ijms24076799 (PMC10095147; doi:10.3390/ijms24076799)
Supplement: Supplementary file 1 [file ijms-24-06799-s001.zip › ijms-2269217-supplementary.pdf]

Supplementary Materials

Detailed Protocol

1. Lentivirus Production and Titration

1.1 Prepare 100 mm Petri dish of HEK293T line cells with 70-80% of confluency. In order to obtain proper titer we typically use medium containing lentiviral particles collected from 4 10 cm dish per 1 virus. Dilute 2 μg of pMD2 (Addgene, cat. no. 12260), 6 μg of pPAX (Addgene, cat. no. 12259) and 8 μg of lentivirus plasmid of interest in 1 ml of medium without serum (DMEM or opti-MEM) for each plate. Add 48 μl of 1 mg/ml polyethylenimine solution (Polyscience, Cat.№ 23966), vortex thoroughly and incubate for 10 minutes. Add 1 ml of plasmids-PEI mixture to Petri dish with 9 ml of DMEM and incubate HEK293T cells overnight. Next morning aspirate medium, wash plates with warm PBS and add fresh HEK293T media. 48 hours after transfection collect culture medium containing lentiviral particles and centrifuged 5 minutes at 2500 rpm. Then the supernatant was passed through a polyether sulfone (PES) or polyvinylidene fluoride (PVDF) filter with a pore size of 0.45 μm and transferred to a ultracentrifuge tube. We recommend high speed ultracentrifugation at 70 000 g for 2 hours at 4˚C as in original protocol in order to obtain proper virus titer. However, it is also possible to increase the centrifugation time or to use more amount of medium containing lentiviral particles. You can recalculate the optimal centrifugation time based on the parameters the rotor. After centrifugation aspirate supernatant and resuspend the pellet in cold DPBS containing 20% sucrose. Prepare several single use aliquots of concentrated lentiviruses. Immediately froze the aliquots in liquid nitrogen and store at -80˚C.

1.2 The efficiency of direct reprogramming procedure and cell survival is mainly determined by the optimal titer of lentiviruses encoding microRNAs and transcription factors. Perform lentiviruses titration in primary human fibroblasts with appropriated antibiotic selection for each virus. Titration is based on kill curve assay. Titer consider optimal when >95% cells alive after antibiotic selection (based on trypan blue staining).

2. Reprogramming procedure

2.1 Plate primary human dermal fibroblasts on 12 well plate till ≈ 95% confluency.

2.2 Thaw on ice lentiviruses encoding rtTA, lenti-pTight-9-124-BclxL, DLX2 and CTIP2. For each well add appropriate amount of viruses to fibroblast media (1 ml total per each well). Add polybrene 8 μg/ml to the fibroblast media (FM)/lentivirus solution. Apply mixture to each well of the plate.

2.3 The next morning aspirate medium containing lentiviruses, wash the cells twice with PBS (37˚ C) and add fresh media supplemented with 1 μM rapamycin.

2.4 Two days later add DOX (2 μg/ml) to the FM in order to induce microRNA expression.

2.5 Five days after transduction, feed transduced fibroblast with 3 μg/ml puromycin and 1 μg/ml DOX solution for the selection of postmitotic cells. This is the first selection step.

2.6 Seven days after transduction wash cells with warm PBS twice. Then aspirate PBS and add 400 µl per well of 0.25% trypsin-EDTA solution. Wash cells with trypsin-EDTA solution, aspirate and add fresh 100 µl per well of 0.25% trypsin-EDTA solution. Incubate at 37 °C for 3-5 min until cell detach. Once the cells are detached add 2 ml per well FM+1 µg/ml DOX. Transfer cells into sterile centrifuge tube. Wash out the cells from trypsin by centrifugation at 300 g for 3 min at room temperature. Add 500 µl of Fibroblast media+1 µg/ml DOX to the pellet and resuspended cells. Add 940 ul of Fibroblast media+1 µg/ml DOX and apply 50 ul per well as a drop to each coverslip precoated with Matrigel of 24 well plate. Incubate drop-plated plates in a humidified, 5% CO_2, 37 °C incubator until the cells have attached (usually 15-20 minutes) before adding 500 µl per well FM + 1 µg/ml DOX.

2.7 After cells completely attached add appropriate amount of MYT1L lentivirus.

2.8 Next day aspirate media from re-plated cells. Wash 2 times with PBS and add 500 µl per well of reprogramming media (RM) containing 1 µg/ml DOX + 3 µg/ml puromycin+3 µg/ml blasticidin. This step is second selection.

2.9 Repeat the feeding with RNM (500 µl per well) + 1 µg/ml DOX every 5 d. Add 0,5 µg/ml DOX to each well every 2 d between feedings. Continue to feed cells with DOX for at least 30 day starting the day when lentiviruses were added (36-day total). Starting 20 day after transduction start to remove 250 ul per well of old media before adding new one every 5 day.

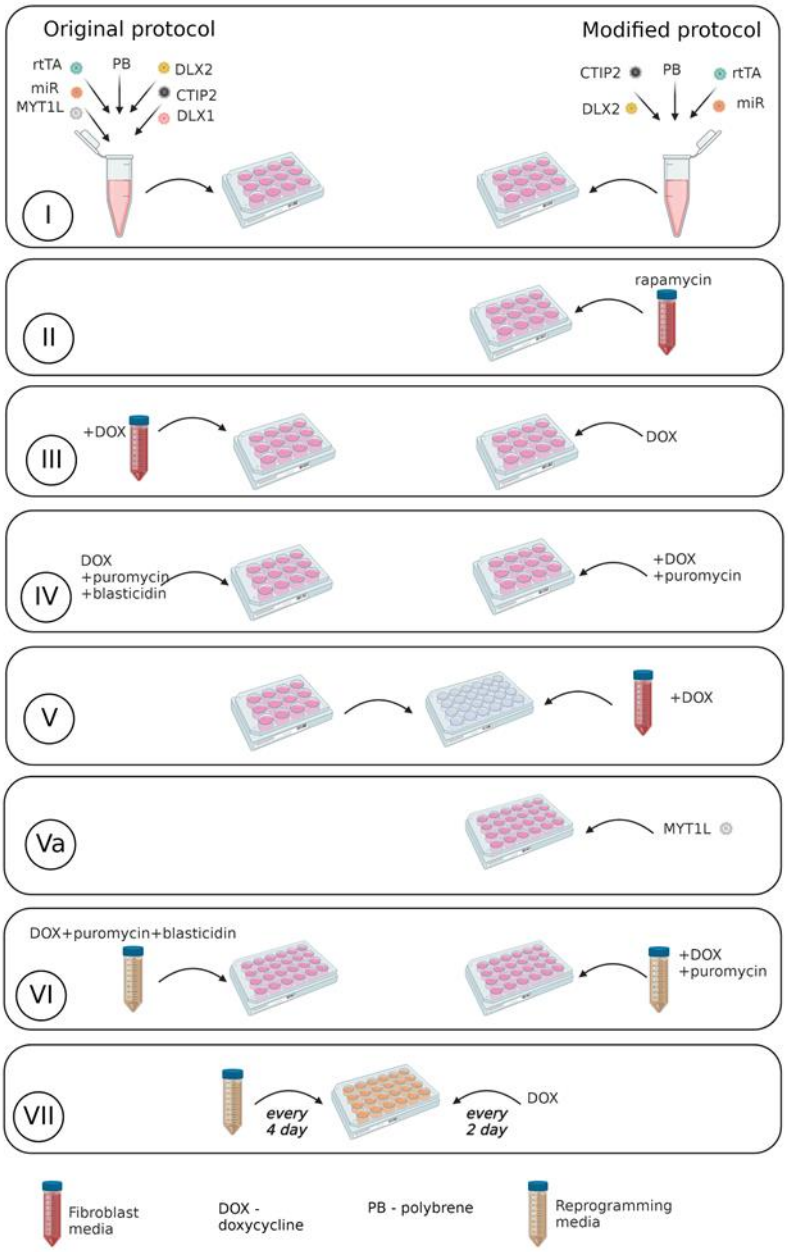

Figure S1. (Related to Figure 1). Schematic representation of the main steps of the original and modified protocol. Created with BioRender.com.
